# Evaluation of tools for long read RNA-seq splice-aware alignment

**DOI:** 10.1093/bioinformatics/btx668

**Published:** 2017-10-23

**Authors:** Krešimir Križanović, Amina Echchiki, Julien Roux, Mile Šikić

**Affiliations:** 1 Department of Electronic Systems and Information Processing, Faculty of Electrical Engineering and Computing, University of Zagreb, Zagreb, Croatia; 2 Département d’Ecologie et d’Evolution, Université de Lausanne, Quartier Sorge, Lausanne, Switzerland; 3 Swiss Institute of Bioinformatics, Lausanne, Switzerland; 4 Bioinformatics Institute, Singapore, Singapore

## Abstract

**Motivation:**

High-throughput sequencing has transformed the study of gene expression levels through RNA-seq, a technique that is now routinely used by various fields, such as genetic research or diagnostics. The advent of third generation sequencing technologies providing significantly longer reads opens up new possibilities. However, the high error rates common to these technologies set new bioinformatics challenges for the gapped alignment of reads to their genomic origin. In this study, we have explored how currently available RNA-seq splice-aware alignment tools cope with increased read lengths and error rates. All tested tools were initially developed for short NGS reads, but some have claimed support for long Pacific Biosciences (PacBio) or even Oxford Nanopore Technologies (ONT) MinION reads.

**Results:**

The tools were tested on synthetic and real datasets from two technologies (PacBio and ONT MinION). Alignment quality and resource usage were compared across different aligners. The effect of error correction of long reads was explored, both using self-correction and correction with an external short reads dataset. A tool was developed for evaluating RNA-seq alignment results. This tool can be used to compare the alignment of simulated reads to their genomic origin, or to compare the alignment of real reads to a set of annotated transcripts. Our tests show that while some RNA-seq aligners were unable to cope with long error-prone reads, others produced overall good results. We further show that alignment accuracy can be improved using error-corrected reads.

**Availability and implementation:**

https://github.com/kkrizanovic/RNAseqEval, https://figshare.com/projects/RNAseq_benchmark/24391

**Supplementary information:**

Supplementary data are available at *Bioinformatics* online.

## 1 Introduction

Over the past 10 years, the use of next generation sequencing (NGS) platforms, in particular Illumina, has expanded to dominate the genome and transcriptome sequencing market. Their sequencing-by-synthesis approach is indeed much cheaper and faster than the previously used Sanger sequencing. Recently, two new sequencing technologies—the so-called ‘third generation sequencing technologies’—have emerged, that produce longer reads and hold numerous promises for genomic and transcriptomic studies.

First, the single-molecule sequencing technology unveiled in 2010 by Pacific Biosciences (PacBio), produces reads up to a few tens of thousands of base pairs long. However, raw reads (‘subreads’) display significantly higher error rate (∼10–20%) than reads from the Illumina technology (∼1%) (Schirmer *et al.*, 2015; Ross *et al.*, 2013; Glenn, 2011). To reduce error rates, circularized fragments are sequenced multiple times and the subreads produced can be reconciled to produce higher-quality consensus ‘Reads of Insert’ (ROIs, previously called Circular Consensus Reads). However, there is a trade-off between the ROIs length and accuracy because longer fragments accumulate fewer sequencing passes.

Second, the portable MinION sequencer presented in 2014 by Oxford Nanopore Technologies (ONT), produces even longer reads (up to a few hundreds of thousand base pairs long), but with even higher error rates. Using the R7.3 chemistry, raw reads (‘1 D’ reads) had an error-rate of >25%, while consensus ‘2 D’ reads (where template and complement of double-stranded fragments are successively sequenced and reconciled) displayed 12–20% error rate (Laver *et al.*, 2015; Sović *et al.*, 2016). It is likely that improvement in the chemistries (notably the recently released R9 and R9.4) has reduced error rates (http://lab.loman.net/2016/07/30/nanopore-r9-data-release).

For transcriptomic studies, long reads of these third generation sequencing technologies should be very helpful in the challenging task of identifying isoforms, and estimating reliably and precisely their abundances (Garber *et al.*, 2011; Łabaj *et al.*, 2011). It is unclear though whether high error rates will allow precise identification of exon-exon junctions required for proper discrimination of isoforms that are very similar in sequence (e.g. NAGNAG splicing).

The aim of this work was to determine whether currently available RNA-seq splice-aware aligners could handle third generation sequencing data, namely much longer read length and significantly higher error rate. Such a benchmark of RNA-seq alignment tools and pipelines, previously performed on both real and synthetic Illumina reads (Engström *et al.*, 2013) proved to be very helpful for the community of end-users. Another benchmark of RNA-seq alignment tools was performed on synthetic data of varying error rate and complexity (Baruzzo *et al.*, 2017). However, to the best of our knowledge, no tests were performed on third generation sequencing data.

Splice-aware RNA-seq alignment tools can be divided into two groups. First, guided splice-aware aligners, use the genome sequence and known gene annotations to calculate gene or transcript abundance, but cannot be used to identify new splice junctions. Second, de novo splice-aware aligners can align RNA-seq reads to a reference genomic sequence without prior information on gene annotations.

BBMap is to our knowledge the only tool explicitly claiming support of both PacBio and ONT reads (Bushnell *et al.*, 2014). It uses short k-mers to align reads directly to the genome, spanning introns to find novel isoforms. It uses a custom affine-transform matrix to generate alignment scores.

A tutorial, developed by the PacBio team (available at https://github.com/PacificBiosciences/cDNA_primer/wiki/Aligner-tutorial:-GMAP,-STAR,-BLAT,-and-BLASR) recommends modified sets of parameters for the alignment of PacBio reads with STAR and GMAP, based on in-house testing. STAR (Dobin *et al.*, 2013) employs sequential maximum mappable seed search in uncompressed suffix arrays followed by seed clustering and stitching procedure. It detects novel canonical, non-canonical splices junctions and chimeric-fusion sequences. GMAP (Wu *et al.*, 2016) is a part of GMAP/GSNAP package and uses diagonalization to find exon regions, oligomer chaining of short k-mers to refine them, and dynamic programming at the nucleotide level to resolve mismatches, indels and intron boundaries.

In our tests we included TopHat2 (Kim *et al.*, 2013), the most popular aligner for Illumina reads. TopHat2 implements a two-step approach where initial read alignments are first analyzed to discover exon-exon junctions, which are then used in the second step to determine the final alignment. HISAT2, the successor of Tophat2, was also included. It uses a global FM-index, as well as a large set of small FM-indexes (called local indexes) that collectively cover the whole genome. This strategy enables effective alignment of RNA-seq reads spanning multiple exons (Kim *et al.*, 2015).

In the event that aligners are unable to cope with high error rates in the reads, we tested if the addition of an error-correction step before the mapping step could be useful. Recent tools have been developed that allow error correction of reads from third generation sequencing technologies, taking advantage of the redundancy within each dataset, or combining them with second generation sequencing datasets (Bradley *et al.*, 2012). The latter (so-called ‘hybrid’) approach has already been used to obtain a comprehensive characterization of the transcriptome of the human embryonic stem cell (Au *et al.*, 2013). In this study, we applied both approaches and quickly discuss their merits.

## 2 Materials and methods

Since the actual origin of reads in real datasets is unknown and can only be estimated through the alignment process, real datasets are not best suited to assess the performance of alignment tools. The accuracy and precision of aligners can be assessed on synthetic data, but in return simulators fail to mimic every aspect of real-life datasets, potentially biasing the benchmark results. In this study, we thus decided to use both simulated and real datasets.

All real datasets consist of RNA converted to cDNA and amplified prior to sequencing. For simulation, we have used the PacBio reads simulator PBSIM (Ono *et al.*, 2013). Several datasets were simulated with different parameters, and using the annotated transcriptome of different organisms (the baker’s yeast *Saccharomyces cerevisiae*, the fruit fly *Drosophila melanogaster*, and human chromosome 19; see Supplementary Material).

To more precisely explore subpar performance of some mappers tested in this study, we simulated a dataset with long reads containing very few errors. This allows us to estimate whether a mapper performs poorly because of longer reads or because of higher error rate.

The focus in our tests was on PacBio technology, for which we had a large amount of real data and a dedicated simulator (PBSIM). However, we also included one real ONT dataset. For comparison, one ONT MinION dataset was also simulated on *Drosophila melanogaster* using PBSIM, setting the parameters according to the statistics of ONT MinION R9 real data. While a PacBio simulator is not entirely appropriate for ONT MinION data, we felt that mimicking their read length and error profile (frequency of insertions, deletions and mismatches) should provide some useful insight. At the time of our simulation experiments, we were unaware of a dedicated MinION reads simulator. Since then, we became aware of NanoSim (Yang *et al.*, 2017), but due to time constraints decided not include it in our benchmark.

Additional synthetic ONT MinION dataset was simulated using human chromosome 19. Results are like those achieved on the first ONT MinION simulated dataset and are presented in Supplementary Note S4.

In order to explore the effect of read error correction on alignment, the highest quality real PacBio dataset was error corrected using the recent consensus tool Racon (Vaser *et al.*, 2017). Both correction using external Illumina reads and self-correction were explored.

The description of simulated datasets generation can be found in the Supplementary Material. Table 1 shows relevant statistics of test datasets. As can be seen from the table, datasets vary in size and complexity. For example, datasets 2 and 4 have similar size because they were generated using the same approximation of the gene coverage histogram, however, since MinION ONT reads are on average longer than PacBio reads, dataset 2 contains more reads than dataset 4.

**Table 1.** Test dataset statistics

Data set	Type	Organism	Technology	Size	No. genes	No. reads	% AS genes
A	Real	*D. melanogaster*	Illumina	1 GB	NA	4,000,000	NA
B	Synthetic	*D. melanogaster*	Long read low error	1.4 GB	7,000	410,000	10
1	Synthetic	*S. cerevisiae*	PacBio ROI	400 MB	6,000	185,000	0
2	Synthetic	*D. melanogaster*	PacBio ROI	1.4 GB	7,000	412,000	10
3	Synthetic	*Homo sapiens, chr. 19*	PacBio ROI	200 MB	1,520	84,000	60
4	Synthetic	*D. melanogaster*	ONT R9 2D	1.4 GB	7,000	342,000	10
5	Real	*D. melanogaster*	PacBio ROI	1 GB	NA	192,000	NA
6	Real	*D. melanogaster*	PacBio ROI error-corrected	500 MB	NA	192,000	NA
7	Real	*D. melanogaster*	PacBio Subreads	1 GB	NA	243,000	NA
8	Real	*D. melanogaster*	ONT R9 2D	120 MB	NA	40,000	NA

All the data used to create test datasets (and the datasets themselves) is available through FigShare (https://figshare.com/projects/RNAseq_benchmark/24391).

### 2.1 Datasets

To generate simulated datasets, we used PBSIM version 1.0.3, downloaded from https://code.google.com/archive/p/pbsim/.

Synthetic datasets were created from the following organisms:

- *Saccharomyces cerevisiae* S288 (baker’s yeast)
- *Drosophila melanogaster* r6 (fruit fly)
- *Homo Sapiens* GRCh38.p7 (human)

Reference genomes for all organisms were downloaded from http://www.ncbi.nlm.nih.gov.

PBSIM is intended to be used as a genomic reads simulator, taking as input a reference sequence and a set of simulation parameters (e.g. coverage, read length, error profile). To generate RNA-seq reads, PBSIM was applied to a set of transcripts generated from a particular genome using the gene annotations downloaded from https://genome.ucsc.edu/cgi-bin/hgTables. To make the datasets as realistic as possible, real datasets were analyzed and used to determine simulation parameters. Real gene expression datasets were used to select a set of transcripts for simulation (downloaded from http://bowtie-bio.sourceforge.net/recount/; core (human), nagalakshmi (yeast) and modencodefly (fruit fly) datasets were used) (Frazee *et al.*, 2011).

A detailed description of the whole process used to simulate synthetic data is given in Supplementary Note S1.

Real RNA-seq datasets used in this benchmark were generated from *D. melanogaster*. Technical replicates of the same sample were sequenced with three different technologies: Illumina HiSeq, PacBio RSII and ONT MinION. Illumina data were used for baseline comparison of all tested tools and for error correction of PacBio reads. PacBio and MinION data were used to assess the aligners’ performances and to determine error profiles that were then used for simulation of synthetic data. In total, we used:

- 1GB of Illumina reads, subsampled randomly from a larger size dataset. Reads were of size 101 bp. Illumina data was included just as a baseline, to show that all tools work rather well on Illumina, and to use them for error correction. Because of that, we used Illumina reads without paired-end information.
- Over 5GB of PacBio subreads, sequenced from three different size fractions of transcripts (1–2 kb, 2–3 kb and 3–7 kb, 2 SMRT-cells sequenced for each size fraction). This corresponded to about 2GB of Reads of Insert extracted from the subreads.
- 350MB of ONT MinION reads using the R9 chemistry. Because of the very low quality of 1 D reads, only 2 D reads were used in this benchmark.

### 2.2 Error correction

To test if the alignment results could be improved using error correction, the highest quality PacBio dataset (containing ROIs) was corrected. Error correction was performed using Racon (Vaser *et al.*, 2017). Correction using Illumina reads, and self-correction were tested. Since self-corrected dataset proved to have better error profile, only this dataset was retained for the benchmark (Dataset statistics is given in Supplementary Table S1).

Supplementary Table S1 displays error rate and read length statistics for all real datasets, including all datasets obtained using error correction.

### 2.3 Evaluated RNA-seq tools

We tested five RNA-seq alignment tools that have been updated recently reflecting that they are still being maintained.

#### *2.3.1* STAR

Downloaded from https://github.com/alexdobin/STAR. Version 2.5.2b was used. On Illumina dataset STAR was run using regular script STAR, while on long read datasets STAR was run using the STARlong script with parameters suggested at Bioinfx study: Optimizing STAR aligner for Iso-Seq data from PacBio GitHub pages (https://github.com/PacificBiosciences/cDNA_primer/wiki/Bioinfx-study:-Optimizing-STAR-aligner-for-Iso-Seq-data).

#### *2.3.2* Tophat2

Binaries were downloaded from https://ccb.jhu.edu/software/tophat/index.shtml and used with Bowtie2. Version 2.1.1 was used, with default parameters for alignment. SAMTools version 1.2 were used to convert Tophat output from BAM to SAM format.

#### *2.3.3* Hisat2

Binaries were downloaded from https://ccb.jhu.edu/software/hisat2/index.shtml. Version 2.0.4 was used, with default parameters for alignment.

#### *2.3.4* BBMap

Downloaded from https://sourceforge.net/projects/bbmap/. The script mapPacBio.sh was used. BBMap version 35.92 was used. Reads were first converted to FASTA format (originally in FASTQ format) using samscripts tool (https://github.com/isovic/samscripts). The program was then run with the option *fastareadlen* set to a value appropriate for each dataset.

#### *2.3.5* GMAP/GSNAP

Source code was downloaded from http://research-pub.gene.com/gmap/. Version 2016-11-07 was used. GMAP was used with default parameters, as recommended in the tutorial for using GMAP with PacBio data (https://github.com/PacificBiosciences/cDNA_primer/wiki/Aligner-tutorial%3A-GMAP%2C-STAR%2C-BLAT%2C-and-BLASR).

We also ran GSNAP on Illumina dataset (since it is tailored for short reads), but with default parameters and without paired-end information it mapped slightly less reads then GMAP and we decided not to use it.

Exact commands used to run each tool can be found in Supplementary Note S2.

### 2.4 RNAseqEval tool

Three of the five RNA-seq aligners were evaluated on resource usage and alignment quality. CPU and memory consumption were evaluated using a fork of the Cgmemtime tool (https://github.com/isovic/cgmemtime.git).

To evaluate the quality of each aligner, we developed RNAseqEval (https://github.com/kkrizanovic/RNAseqEval), meant to be a general tool for evaluating RNA-seq alignments. It is written in Python and contains two main scripts, one for evaluating data simulated using PBSIM and the other for evaluating real data or data whose origin is unknown. Both scripts require aligner output in SAM format which they compare to gene annotations and, in case of simulated data, alignment files in MAF format describing the origin of each simulated read.

#### *2.4.1 *Evaluating synthetic data

The script for evaluating synthetic or simulated data currently works only on data simulated with PBSIM, but could be expanded in the future to support other simulators. Aside from aligner output in SAM format and gene annotations in GTF or BED format, the script takes a folder containing files generated by PBSIM. The folder containing PBSIM data needs to have a specific structure and follow a specific naming convention, as described in the program documentation.

For each read from aligner output, the script will use PBSIM generated MAF files and gene annotations to find its origin on the reference genome and will compare it to the alignment calculated by the aligner. The start and end position of an alignment and of read origin are compared, and an error of five nucleotides is tolerated. The script outputs summary information on how many reads were accurately aligned to their chromosome, strand and position of origin.

#### *2.4.2* Evaluating real data

The script for evaluating real data takes only aligner output in SAM format and gene annotation in GTF or BED format as its input. Because the origin of a read is unknown, the script will check annotations for genes with which the read overlaps, and then evaluate how well a read alignment matches exons and introns of that gene.

When matching beginning and end of an alignment to each exon in an annotation, an error of five nucleotides is tolerated. Similarly, an overlap between an alignment and an exon annotation needs to be at least five base-pairs to be considered valid. We tested different values for allowed error (and minimum overlap) and increasing it above five base-pairs did not noticeably improve the results.

## 3 Results

### 3.1 Baseline comparison

We first examined how alignment tools performed on the Illumina ‘baseline’ dataset A (Table 2). We found that all aligners managed to align a large fraction of Illumina reads.

**Table 2.** Percentage of reads aligned over all aligners and datasets

Data set	Aligner	Tophat2 (%)	Hisat2 (%)	STAR (%)	BBMap (%)	GMAP (%)
	No. reads					
A	4M	85.2	94.8	96.8	**97.6**	96.7%
B	410K	0	0	84.9	97.3	**99.9**
1	185K	0.7	6.77	48.9	**91.4**	89.2
2	412K	0	0	33.3	84.5	**92.0**
3	84K	0	0	32.3	64.3	**88.3**
4	342K	0	0	5.5	43.0	**98.8**
5	192K	0	0	46.1	74.5	**85.4**
6	192K	0	0.4	67.2	82.8	**88.5**
7	243K	0	0%	0.1	72.8	**89.7**
8	40K	0	0%	16.7	88.0	**98.3**

*Note*: Bold values present the best scoring result for a particular measured value.

On datasets that include longer and more erroneous reads however (dataset 1 to dataset 8), there were large discrepancies across tools. In particular, Tophat2 and Hisat2, with default parameters, aligned <7% of the reads for all long-read datasets. To be fair, it has to be stated they do not claim to work with long-reads and were included in the test for the sake of completeness. Therefore, we did not consider these two tools in further analyses, and we focused on the remaining three aligners: BBMap, GMAP and STAR.

If we look at the results dataset B (long reads with low error), we can see that Tophat2 and Hisat2 fail to align almost any reads using default parameters (the number in the table are rounded down). We can conclude that Tophat2 and Hisat2 are tailored for short NGS reads and are not able to handle longer read lengths.

Based on the percentage of reads aligned, the best results were achieved by GMAP, which aligned >85% of reads across the all tested datasets.

BBMap performed slightly better on Illumina (dataset A) and on synthetic *S. cerevisiae* PacBio dataset (dataset 1, which contains very few multi-exon transcripts), but the fraction of reads aligned fell behind GMAP on more complex synthetic datasets and on real datasets (e.g. only 43% of the synthetic *H. sapiens* PacBio reads of dataset 4 were aligned).

STAR managed to align a large percentage of Illumina reads (96.8%), but its performance was uneven across third generation sequencing datasets, aligning from 0.1% to 67.2% of the reads, and often aligning less than half of the reads. STAR was seemingly affected by increased complexity of the datasets, as well as by increased error rates (Illumina and error-corrected PacBio datasets achieving the best performance). Since STAR managed to align a significant portion of dataset B (long reads low error), we can conclude that it can handle long reads, but has trouble with higher error rates especially on more complex datasets.

Across all tools, error correction improved alignment rates, as can be seen from the comparison of dataset 5 and dataset 7.

In summary, for some aligners the percentage of alignment for third generation sequencing technologies reads was similar to what is achieved for Illumina reads. However, looking only at the number of the reads each tool managed to align to a genome is not a reliable measure of general alignment quality. For example, a tool could align most of the reads, but only on only a portion of their length, or it could align them at incorrect location on the genome.

### 3.2 Synthetic datasets

**Fig. 1.**
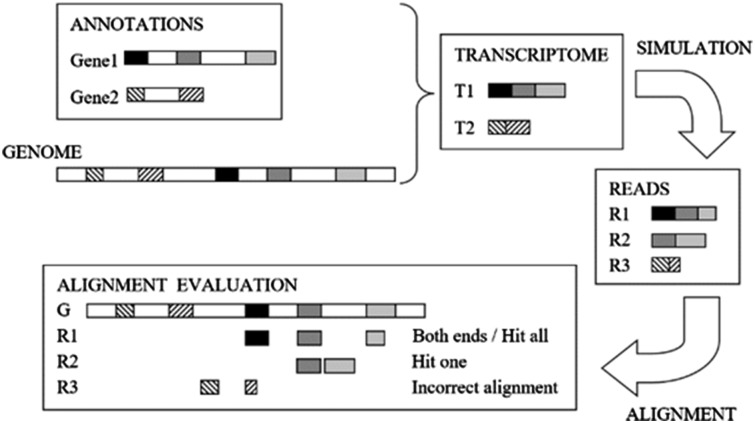
Evaluation of synthetic datasets

To get more insights into the quality of the alignments, we evaluated the aligners on four synthetic datasets generated from transcriptomes of varying complexity using the PBSIM tool (Materials and methods), and supposed to reflect characteristics of the PacBio (datasets 1–3) and ONT MinION technologies (dataset 4). In these datasets, the precise origin of each read is known, allowing to assess the alignment quality by examining how well the alignment location matches the origin location in the genome. The alignment results for those datasets were evaluated using the RNAseqEval tool, as summarized in Figure 1. 

All results are displayed as the percentage of all reads in the dataset. The percentages of reads that were aligned is shown (without assessing the accuracy), the match rate of aligned reads, percentage of reads for which the beginning, the end and inner exon boundaries are accurately placed within five base-pairs (Correct), percentage of reads that overlap all exons of the read origin (Hit all) and percentage of reads that overlap at least one exon of the read origin (Hit one). Match rate is calculated as a percentage of aligned bases that are equal to the corresponding bases on the reference. Overlaps of hit one and hit all statistics need to be at least five bases.

Results of the evaluation on all synthetic reads are shown in Table 3. The evaluation on the subset of split reads (i.e. reads aligned to multiple non-contiguous locations on the reference genome) is also shown. Split reads, if aligned correctly, should overlap at least one exon-exon junction in the transcript of origin, and thus cover two or more exons. Percentages of reads shown in Table 3 are relative to the number of reads in input; the percentage relative to the number of aligned reads are shown in Supplementary Table S2.

**Table 3.** Aligner evaluation on synthetic datasets

Dataset		STAR (%)	BBMap (%)	GMAP (%)
1	Aligned	48.9	**91.4**	89.2
	Match rate	**93.7**	92.5	92.3
	Correct	22.1	**48.2**	41.8
	Hit all	46.5	**87.0**	84.3
	Hit one	47.1	**88.1**	85.4
	Split reads	1.89	**3.46**	3.3
	Correct, split	0.55	**1.1**	0.95
	Split hit all	1.2	**2.2**	2.05
	Split hit one	1.8	**3.3**	3.1
2	Aligned	33.3	84.5	**92.0**
	Match rate	**94.0**	89.9	92.0
	Correct	10.4	24.9	**30.3**
	Hit all	27.7	54.4	**73.1**
	Hit one	30.7	78.4	**85.4**
	Split reads	23.9	64.8	**72.8**
	Correct, split	6.3	14.2	**21.6**
	Split hit all	19.3	36.7	**56.1**
	Split hit one	22.3	60.7	**68.5**
3	Aligned	32.3	64.3	**88.3**
	Match rate	**94.3**	86.2	91.8
	Correct	11.4	15.3	**28.0**
	Hit all	27.5	26.8	**70.0**
	Hit one	30.5	61.2	**83.7**
	Split reads	23.1	46.0	**70.0**
	Correct, split	7.5	4.3	**19.9**
	Split hit all	19.4	10.2	**54.1**
	Split hit one	22.4	44.5	**68.0**
4	Aligned	5.5	43.0	**98.8**
	Match rate	89.6	88.4	**90.5**
	Correct	1.2	7.9	**22.8**
	Hit all	5.0	26.8	**87.1**
	Hit one	5.3	42.1	**97.1**
	Split reads	3.2	34.2	**80.7**
	Correct, split	0.5	4.1	**16.2**
	Split hit all	2.9	18.7	**70.0**
	Split hit one	3.2	33.8	**79.8**

*Note*: Bold values present the best scoring result for a particular measured value.

Overall, the most accurate alignments were given by GMAP, followed by BBMap and with STAR being worse than the other two. The exception is dataset 1, on which BBMap proved slightly better than GMAP. On datasets 2, 3 and 4 GMAP surpasses other two tools in both mapping reads to correct general genomic location (Hit all and Hit one) and in correctly determining their exact position of origin (Correct).

Reads aligned by STAR mostly aligned to correct general genomic locations (hit all and hit one), and displayed very good match rates, however, the low fraction of reads overall aligned (Tables 3 and 4) did not allow this tool to compare favorably to GMAP and BBmap. Moreover, STAR did not perform particularly well at correctly aligning the beginning and end of reads.

**Table 4.** Aligner evaluation on real datasets

Dataset		STAR	BBMap	GMAP
5	Aligned (%)	46.1	74.5	**85.4**
	Match rate (%)	**92**	71	88
	No. expressed genes	8884	9536	**11034**
	Exon hit (%)	45.7	73.4	**83.3**
	Contiguous alignment (%)	33.1	48.4	**54.2**
6	Aligned (%)	67.2	82.8	**88.5**
	Match rate (%)	**93**	72	92
	No. expressed genes	8515	9724	**10641**
	Exon hit (%)	65.1	81.8	**87.0**
	Contiguous alignment (%)	35.0	55.6	**65.1**
7	Aligned (%)	0.1	72.8	**90.1**
	Match rate (%)	81	68	**82**
	No. expressed genes	183	9013	**11046**
	Exon hit (%)	0.1	72.4	**86.0**
	Contiguous alignment (%)	0.0	35.7	**41.6**
8	Aligned (%)	16.8	88.0	**98.3**
	Match rate (%)	**83**	67	81
	No. expressed genes	2344	6578	**7224**
	Exon hit (%)	11.0	62.3	**68.8**
	Contiguous alignment (%)	4.8	26.8	**30.5**

*Note*: Bold values present the best scoring result for a particular measured value.

Datasets 2, 3 and 4 contain a significant number of split reads. Focusing on split read statistics on those datasets, BBMap performed significantly worse than GMAP and sometimes than STAR: on dataset 3 it managed to overlap all exons from a read origin (Split hit all) less precisely than STAR (10.2% versus 19.4%). For STAR, results for split reads were in line with its overall results, but the overall number of aligned reads being so low, STAR cannot be recommended for the alignment of third generation sequencing RNA-seq reads.

Overall, BBMap outperformed GMAP in alignment precision on dataset 1 with lower complexity (less multi-exon genes), but lagged behind in general alignment efficiency, sometimes by a large margin, on more complex datasets. This indicates that BBMap should be used with caution to align split RNA-seq reads. In this setting, GMAP shows the best performance and should be preferred, although the results on dataset 1 indicate that it still has some room for improvement in dealing with high error rates of third generation sequencing data.

### 3.3 Real datasets

For real data, the origin of each read is not known, thus aligners were evaluated by comparing the read alignment locations to a given set of gene annotations. Some other relevant statistics, such as alignment match rate and number of expressed genes, were also extracted (Table 4). Percentages of reads shown in Table 4 are relative to the number of reads in input. Supplementary Table S3 also shows percentages of reads relative to the number of reads aligned.

The table shows percentage of reads that were aligned (without assessing the accuracy), percentage of reads that overlap at least one exon (exon hit) and percentage of reads that overlap one or more exons in a sequence, corresponding to a gene annotation (contiguous exon alignment). All values are displayed as the percentage of all reads in the dataset. The table also shows the number of expressed genes and average match rate of aligned reads. Match rate is calculated as a percentage of aligned bases that are equal to the corresponding bases on the reference. Overlaps for exon hit statistics need to be at least five bases.

All real datasets consisted of technical replicates of RNA-seq on the same D. melanogaster sample sequenced on different platforms. Interestingly, these datasets were characterized by different error profiles (Supplementary Table S1).

As expected from previous tests, GMAP showed the best results, followed closely by BBMap. GMAP was slightly better at aligning reads to annotated exonic locations in the genome. The match rate of aligned reads was roughly equal to the determined error profile for each dataset (Shown in Supplementary Table S1) thus suggesting that the reads are aligned to correct positions. GMAP was even able to align ONT MinION data with a reasonable accuracy. It is interesting to note that by some criteria GMAP shows better results on lesser quality dataset 7 (consisting of subreads) compared to higher quality dataset 5 (consisting of ROI) and dataset 6 (error corrected ROI).

Both BBMap and GMAP reported a large percentage of ONT MinION reads aligned, however, match rate and exon hit percentage were lower than for PacBio datasets, indicating that a larger percentage of those alignments were at an incorrect position.

STAR showed the worst alignment results. Reads successfully aligned displayed a high match rate, which might reflect the fact that STAR is unable to align reads with highest error rates, or that alignment settings are very conservative.

Supplementary Table S1 shows that error correction somewhat improved the error profile, increasing average match rate by 2–3%. However, even that slight improvement resulted in visibly better alignment results on dataset 6 for all aligners: more reads reported as aligned, more exons hit, more genes expressed and higher match rate. As shown in Table 4, STAR benefits the most from error correction, BBMap somewhat less and GMAP benefits the least. The conclusion that can be drawn from this is that GMAP is the most tolerant to errors, followed by BBMap with STAR being the least tolerant. This is supported by the results on ‘long read low error’ dataset B shown in Table 2.

**Fig. 2.**
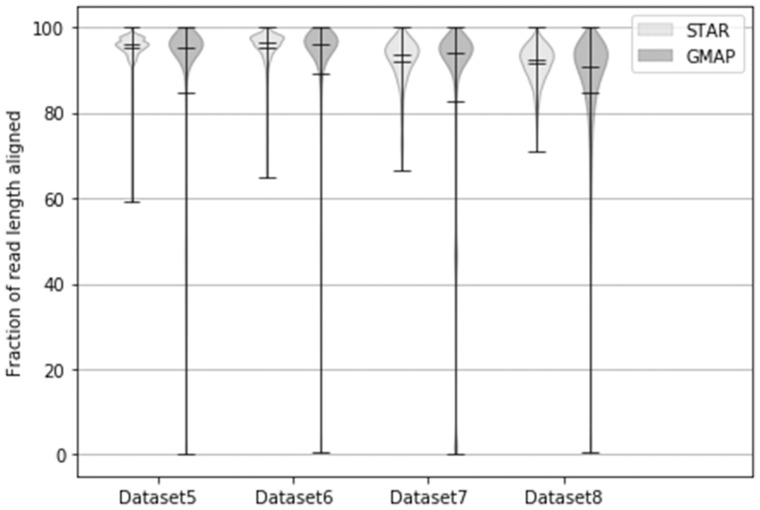
Aligned read percentage violin plots for GMAP and STAR

Finally, we examined what fraction of the read length was aligned (Fig. 2). The results are consistent with other measures of mapping quality, with STAR on average managing to align reads on a slightly larger portion of their length compared to GMAP. BBMap results are not displayed because in the tested settings, all alignments are made on the whole length of the reads (global alignments). This makes the violin plots in Figure 2 for BBMap useless because each read is aligned along 100% of its length. This behavior has some implication in the reported results, as the alignment on both ends of the reads is sometimes incorrect, resulting in lower match rates. It could be a good idea to clip alignments resulting from BBMap, for example using the ‘local’ flag, which converts global alignments into local alignments by clipping them if that results in higher scores. 

### 3.4 Resource usage

To estimate the efficiency of each RNA aligner, CPU time and Maximum memory usage (Resident set size - RSS) were measured. All tools were run in a multithreaded environment, on 12 threads where possible, and total CPU time was measured. The results are shown in Supplementary Figure S1. Illumina data (dataset A) and long read low error data (dataset B) were omitted from this analysis because the focus of the paper is on third generation sequencing data.

Running time seemed to depend on dataset size. In all settings, GMAP used the least amount of memory and ran the fastest. STAR was the slowest and consistently used 60–80 GB of RAM. BBMap memory footprint was also consistently around 10–15 GB of RAM.

## 4 Conclusion

In recent years, third generation sequencing devices have been steadily establishing themselves in genomic research. These technologies promise to solve problems caused by the short read length of the NGS. Regarding RNA-seq analysis, longer reads should notably improve transcript identification. However, third generation sequencing technologies also introduce new bioinformatics challenges, mostly due to their high error rate.

In this study, we attempted to assess the ability of currently available RNA-seq alignment tools to work with third generation sequencing data. Five alignment tools were tested using real and synthetic datasets.

Hisat2 and Tophat2 were unable to align almost any read. STAR displayed only passable results on the least erroneous datasets, but failed almost completely on highly error-prone ONT MinION data.

BBMap, performed quite well, especially on PacBio ROI reads (which have lower error rates) and on simpler organisms with less multi-exonic genes. This seems to indicate that although it is a splice-aware aligner, BBMap best performance is achieved on contiguous alignments (e.g. coming from DNA-seq), and might not be best suited for RNA-seq data.

Finally, GMAP showed the best alignment results. It ran the fastest, used the least memory and usually produced the highest alignment rates, especially on complex datasets. BBMap outperformed GMAP only on low complexity simulated dataset which contained very few split reads, which could indicate that although GMAP outperformed other aligners by a significant margin, it still has some room for improvement.

GMAP particularly stands out on dataset 4 containing simulated ONT MinION reads based on wine fly genome. GMAP maps over 97% to an approximately correct position overlapping at least one exon from the read origin, while second best aligner (BBMap), manages to map <50%. The difference in mapping quality is much smaller on real ONT MinION dataset (dataset 8) and on ONT MinION dataset simulated on human chromosome 19 given in Supplementary Note S4.

Overall, aligning third generation sequencing RNA reads is currently viable with some available tools (namely GMAP and BBMap), but we were surprised by the low precision on alignment location. Apart from dataset 1, containing predominately single-exon reads, the best aligner (GMAP) attributed between 20% and 31% of reads to their correct position of origin (±5 bases). It is not clear if this result is inherent to the high error rates of the technologies, or if it is due to alignment algorithms that were not originally developed for these types of data, or to the specific parameters used in this benchmark. For example it would be interesting to test the effects of clipping BBMap alignments on its overall performance.

There is probably large room for improvement, by developing new more sophisticated and more sensitive algorithms, or by incorporating an error-correction step in bioinformatics pipeline before read alignment, since in our tests this visibly improved the alignment results.

## Funding

This work has been supported in part by Croatian Science Foundation under the project UIP-11-2013-7353 ‘Algorithms for Genome Sequence Analysis’. We acknowledge support from the Marie Curie IOF fellowship 273290 to J.R.

*Conflict of Interest:* M.S. has received a complimentary place at an Oxford Nanopore Technologies organized symposium (free registration fee).

## Supplementary Material

Supplementary Data
