# Supplementary material for: Evaluation of tools for long read RNA-seq splice-aware alignment
Source: Bioinformatics. 2017 Oct 23;34(5):748–54. doi: 10.1093/bioinformatics/btx668 (PMC6192213; doi:10.1093/bioinformatics/btx668)
Supplement: Supplementary Data [file btx668_evaluation_of_tools_for_long_read_rnaseq_slice-aware_alignment_supplement.pdf]

Evaluation of tools for long read RNA-seq splice-aware alignment

Supplement

Supplement table 1. Dataset statistics.

For the purpose of the test, error profiles of all real datasets were determined, by aligning them to the corresponding transcriptome using GraphMap (https://github.com/isovic/graphmap) and by running the script errorrates.py from https://github.com/isovic/samscripts. Due to low quality of alignment of ONT Minion RNA reads to the transcriptome, an additional dataset of ONT MinION R9 DNA reads was downloaded from http://lab.loman.net/2016/07/30/nanopore-r9-data-release, aligned to the genome and the error rate was determined using the script mentioned above.

Dataset	Match rate				Read length				Error type ratio
	Mean	Std	Min	Max	Mean	Std	Min	Max	Mismatch:insertion:deletion
0.1	90%	16%	1%	99%	101	0	101	101	100:0:0
5	86%	11%	0.6%	99.9%	3080	2211	46	40305	47:38:15
6a1	89.6%	9.9%	0.6%	99.9%	2734	1587	68	32942	64:16:20
6a2	86%	11%	0.6%	99.9%	3067	2222	54	40304	45:38:17
6b1	89.6%	11%	0%	99.9%	3179	2243	110	40304	68:19:13
6b2	88.4%	11%	0%	99.9%	2955	2149	54	40304	60:27:13
7	85%	9%	2.1%	99.6%	2374	1641	98	36125	36:41:23
8	84%	11%	6%	99%	1567	889	218	11499	52:18:30
ONT Loman lab	88%	10%	56%	99%	7935	3728	159	49915	33:36:31

The table has four entries for dataset 6 – representing different results of error correction of dataset 5. Error correction was performed twice, once using Illumina reads from dataset 0, and once using self-correction. During the error correction process, only some of the reads are successfully corrected, resulting in a more accurate but smaller dataset. To make the corrected dataset comparable to the original one, error corrected reads were joined with original reads that were not corrected.

- 6a1 – Error correction using Illumina reads, dataset containing corrected reads only
- 6a2 – Error correction using Illumina reads, dataset containing all reads
- 6b1 – Self-correction, dataset containing corrected reads only
- 6b2 – Self-correction, dataset containing all reads

When looking only at corrected reads (datasets 6a1 and 6b1), self-correction and correction using Illumina reads have the same mean match rate. However, self-correction managed to correct a much larger number of reads, so when joined with uncorrected reads (dataset 6b2) it has higher mean match rate compared to correction using Illumina reads. Because of the higher mean match rate, and consequently lower error rate, self-corrected dataset containing both corrected and uncorrected reads (6b2) was chosen for the testing.

Supplement table 2. Aligner evaluation on synthetic datasets – precision**.**

Table 3 in the main part of the paper displays as results as a percentage of the total number of reads, representing a recall value for each measure. In here, the same measurements are displayed as a percentage of aligned number of reads, thus representing precision value.

While looking at recall values, STAR showed significantly worse results than BBMap and GMap, looking at precision values, the results displayed by STAR are comparable and often better than other two aligners.

The statistics for all reads is displayed as the percentage of aligned reads and the statistics for split reads is displayed as a percentage of aligned split reads. The percentages of reads that were aligned is shown (without assessing the accuracy), the match rate of aligned reads, percentage of reads for which the beginning and the end and all inner exon boundaries are accurately aligned (**correct**), percentage of reads that overlap all exons of the read origin (**hit all**) and percentage of reads that overlap at least one exon of the read origin (**hit one**). All metrics are calculated with an allowed error of 5 base-pairs.

**Data set**	**Aligner**	**Aligned**	**Match rate**	**Correct**	**Hit all**	**Hit one**	**Split reads**	**Correct, split**	**Split hit all**	**Split hit one**
**1**	**STAR**	48.9%	**93.7%**	45.1%	95.1%	96.2%	3.87%	29.1%	**64.9%**	95.1%
	**BBMap**	**91.4%**	92.5%	**52.8%**	**95.3%**	**96.5%**	3.79%	**31.8%**	64.1%	**95.3%**
	**GMap**	89.2%	92.3%	46.9%	94.6%	95.8%	3.70%	28.7%	62.3%	95.1%
**2**	**STAR**	33.3%	**94.0%**	31.3%	**83.1%**	92.2%	71.8%	26.6%	**80.9%**	93.6%
	**BBMap**	84.5%	89.9%	29.5%	64.4%	92.8%	76.7%	21.9%	56.6%	93.6%
	**GMap**	**92.0%**	92.0%	**33.0%**	79.4%	**92.9%**	79.2%	**29.7%**	77.0%	**94.0%**
**3**	**STAR**	32.3%	**94.3%**	**35.4%**	**85.2%**	94.5%	71.6%	**32.5%**	**84.0%**	96.9%
	**BBMap**	64.3%	86.2%	23.8%	41.7%	**95.2%**	71.5%	9,3%	22.3%	96.9%
	**GMap**	**88.3%**	91.8%	31.7%	79.1%	94.7%	79.3%	28.4%	77.2%	**97.1%**
**4**	**STAR**	5.50%	89.6%	21.7%	**91.5%**	95.7%	58.0%	15.2%	**91.5%**	99.1%
	**BBMap**	43.0%	88.4%	18.4%	62.4%	98.0%	79.6%	12.0%	54.5%	98.9%
	**GMap**	**98.8%**	**90.5%**	**23.1%**	88.2%	**98.2%**	81.7%	**20.1%**	88.6%	98.9%

Supplement table 3. Aligner evaluation on real datasets – recall and precision**.**

Table 4 in the main part of the paper displays as results as a percentage of the total number of reads, representing a recall value for each measure. In here, the table is expanded with the same measurements displayed as a percentage of aligned number of reads, thus representing precision value.

While looking at recall values, GMap displayed clearly the best results of the three aligners tested in detail, looking at precision values the best aligner is not apparent. BBMap manages to hit more exons, but GMap better aligns spliced reads.

The table shows percentage of reads that were aligned (without assessing the accuracy), percentage of reads that overlap at least one exon (**exon hit**) and percentage of reads that overlap one or more exons in a sequence, corresponding to a gene annotation (**contiguous exon alignment**). **Exon hit** and **contiguous exon alignment** values are displayed both as a percentage of the total number of reads (**recall**) and as a percentage of the number of reads aligned (**precision**). The table also shows the number of expressed genes and average match rate of aligned reads. Match rate is calculated as a percentage of aligned bases that are equal to the corresponding bases on the reference.

					**Recall**		**Precision**	
**Data set**	**Aligner**	**Aligned**	**Match rate**	**No. expressed genes**	**Exon hit**	**Contiguous exon alignment**	**Exon hit**	**Contiguous exon alignment**
**5**	**STAR**	46.1%	**92%**	8884	45.7%	33.1%	**99.1%**	**71.9%**
	**BBMap**	74.5%	71%	9536	73.4%	48.4%	98.6%	65.0%
	**GMap**	**85.4%**	88%	**11034**	**83.3%**	**54.2%**	97.6%	63.4%
**6**	**STAR**	67.2%	**93%**	8515	65.1%	35.0%	96.9%	52.1%
	**BBMap**	82.8%	72%	9724	81.8%	55.6%	**98.7%**	67.1%
	**GMap**	**88.5%**	92%	**10641**	**87.0%**	**65.1%**	98.2%	**73.5%**
**7**	**STAR**	0.1%	**81%**	183	0.09%	0.03%	95.8%	27.9%
	**BBMap**	72.8%	68%	9013	72.4%	35.7%	**99.4%**	**49.0%**
	**GMap**	**90.1%**	82%	**11046**	**86.0%**	**41.6%**	95.4%	46.1%
**8**	**STAR**	16.8%	**83%**	2344	11.0%	4.8%	65.8%	28.5%
	**BBMap**	88.0%	67%	6578	62.3%	26.8%	**70.7%**	30.5%
	**GMap**	**98.3%**	81%	**7224**	**68.8%**	**30.5%**	70.0%	**31.0%**

Supplement figure 1. Resource usage

Figure below displays CPU time and Maximum memory usage (Resident set size - RSS) for STAR, BBMap and GMAP on tested datasets. Illumina data (dataset 0.1) and long read low error data (dataset 0.2) were omitted from this analysis because they were not the focus of our testing. The figure shows that GMAP used the least amount of memory and ran the fastest. STAR was the slowest and consistently used 60-80 GB of RAM. BBMap memory footprint was also consistently around 10-15 GB of RAM.

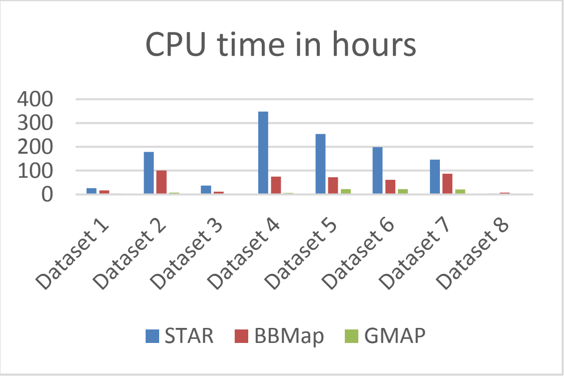

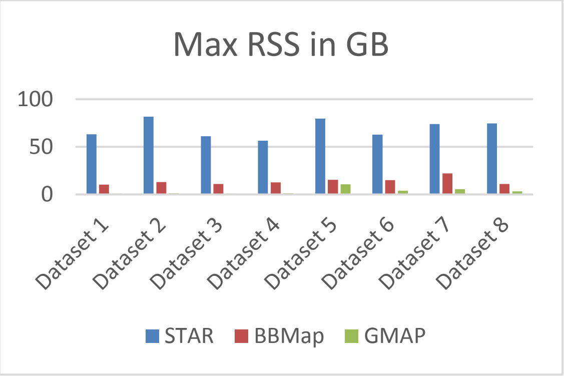

Supplement note 1. Generating synthetic datasets.

To simulate synthetic datasets, we used PBSIM version 1.0.3, downloaded from https://code.google.com/archive/p/pbsim/.

Synthetic datasets were created from the following organisms:

- *Saccharomyces cerevisiae* S288 (baker's yeast)
- *Drosophila melanogaster* r6 (fruit fly)
- *Homo Sapiens* GRCh38.p7 (human)

Reference genomes for all organisms were downloaded from http://www.ncbi.nlm.nih.gov.

PBSIM is intended to be used as a genomic reads simulator, taking as input a reference sequence and a set of simulation parameters (e.g., coverage, read length, error profile). To generate RNA-seq reads, PBSIM was applied to a set of transcripts generated from a particular genome using the gene annotations downloaded from https://genome.ucsc.edu/cgi-bin/hgTables. To make the datasets as realistic as possible, real datasets were analyzed and used to determine simulation parameters. Real gene expression datasets were used to select a set of transcripts for simulation (downloaded from http://bowtie-bio.sourceforge.net/recount/; core (human), nagalakshmi (yeast) and modencodefly (fruit fly) datasets were used).

Simulated data preparation

Simulated datasets were generated using the following workflow:

1. Analyze real datasets to determine error profiles.
2. Filter annotations (keep only primary assembly information) and unify chromosome names.
3. Separate annotations for genes with one isoform and genes with alternative splicing, keeping up to 3 isoforms randomly for each gene with alternative splicing.
4. Generate a transcriptome from processed annotations and a reference genome.
5. Analyze gene expression data and determine gene coverage histogram (see Figure).
6. Approximate gene coverage histogram with 3 points to determine coverage and number of genes in simulated dataset (see Figure). Scale coverages proportionally down to make a smaller dataset, more suitable for testing.
7. Extract 6 subsets of sequences from generated transcriptome, 3 for genes with single splicing and 3 for genes with alternative splicing. Each set contains a number of transcripts corresponding to the number of genes from a previous step.
8. Using *PBSIM*, simulate reads on each generated subset of transcriptome, using coverages determined in step 6 and error profiles determined in step 1.
9. Combine generated reads into a single generated dataset.

For simplicity, we rounded the coverage and number of genes from each transcriptome subset. For example, the Table below shows the numbers used to generate dataset 2 (*D. melanogaster*). The annotation includes roughly 23,000 genes with a single isoform and 3,000 genes with alternative splicing. Rounding up the ratio, we have decided to simulate 1/10 genes with alternative splicing and 9/10 genes without. We considered that each gene undergoing alternative splicing gave rise to three different isoforms with equal expression.

For simulation of PacBio reads, PBSIM parameters (read length, error probability by type, etc) were set to match those of dataset 5 containing reads of insert (see Supplement table 1).

For simulation of MinION ONT reads, *PBSIM* parameters (read length, error probability by type etc.) were set to match those for MinION reads from a R9 chemistry dataset obtained from the Loman lab website (http://lab.loman.net/2016/07/30/nanopore-r9-data-release). Only 2d reads statistics were used.

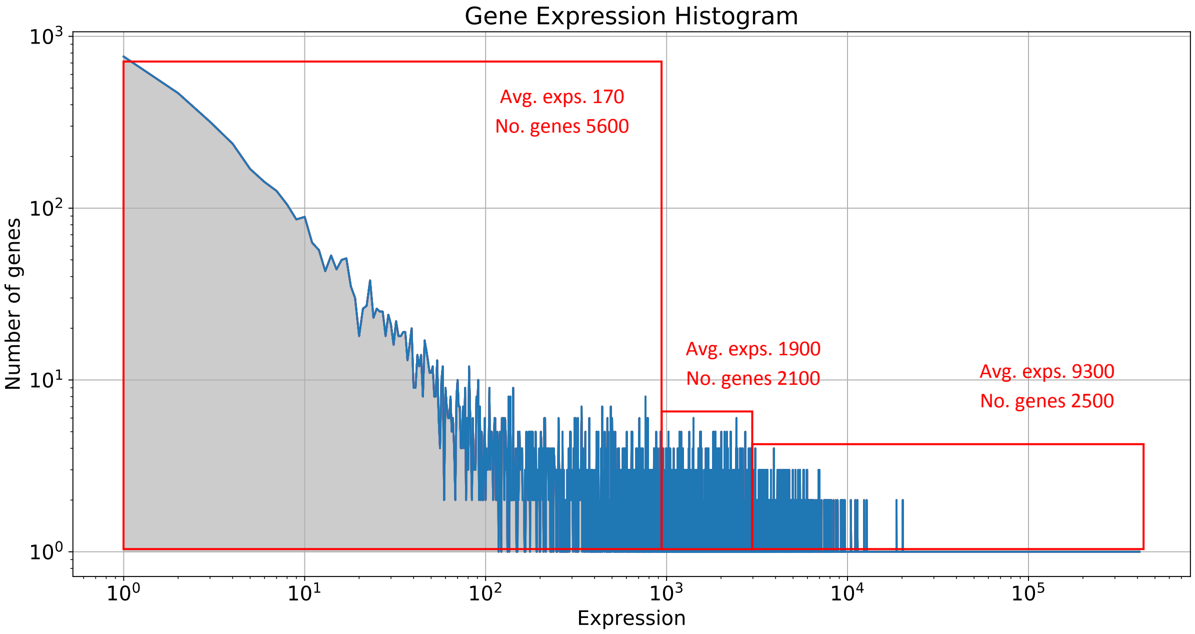

Figure. Data preparation step 6: Approximating gene expression with three points, applied to dataset 2. Three points were chosen as a compromise between achieving simple simulation and realistic datasets.

Table. Generating synthetic dataset 2

Group	Total no. genes	Coverage	Genes without alternative splicing	Genes with alternative splicing	Transcripts with alternative splicing	Coverage for AS transcripts
1	5000	5	4500	500	1500	2
2	2000	50	1750	250	750	15
3	2000	100	1750	250	750	30

Supplement note 2. Commands used to run each tested tools

All tested tools were run with default parameters, except STAR.

STAR:

First, index was created using script STAR:

```
STAR \
--runThreadN 12 \
--runMode genomeGenerate \
--genomeDir results/indexes/staridx_dm \
--genomeFastaFiles drosophila_melanogaster_genome_r6_P.fa
```

Then, STAR was run using script STARlong with the parameters obtained from PacBio GitHub pages: https://github.com/PacificBiosciences/cDNA_primer/wiki/Bioinfx-study:-Optimizing-STAR-aligner-for-Iso-Seq-data

```
STARlong \
--runMode alignReads \
--outSAMattributes NH HI NM MD \
--readNameSeparator space \
--outFilterMultimapScoreRange 1 \
--outFilterMismatchNmax 2000 \
--scoreGapNoncan -20 \
--scoreGapGCAG -4 \
--scoreGapATAC -8 \
--scoreDelOpen -1 \
--scoreDelBase -1 \
--scoreInsOpen -1 \
--scoreInsBase -1 \
--alignEndsType Local \
--seedSearchStartLmax 50 \
--seedPerReadNmax 100000 \
--seedPerWindowNmax 1000 \
--alignTranscriptsPerReadNmax 100000 \
--alignTranscriptsPerWindowNmax 10000 \
--genomeDir results/indexes/staridx_dm \
--readFilesIn test_datasets/dataset5.fastq
```

BBmap:

BBMap will create an index if it does not already exist. It was run with default parameters

```
bbmap/mapPacBio.sh ref= drosophila_melanogaster_genome_r6_P.fa
in=test_datasets/dataset5.fasta out=bbmap_d5.sam
```

TopHat2:

First, index was created using the script bowtie2-build.

```
bowtie2-build drosophila_melanogaster_genome_r6_P.fa tophat2_dm_idx
```

Then, TopHat2 was run with default parameters.

```
tophat2 -o dataset5 tophat2_dm_idx dataset5.fastq
```

Hisat2:

First, index was created using the script hisat2-build.

```
hisat2-build drosophila_melanogaster_genome_r6_P.fa hisat2_dm_idx
```

Then, Hisat2 was run with default parameters.

```
hisat2 -x hisat2_dm_idx -U dataset5.fastq -S hisat2_d5.sam
```

GMAP:

First, index is created using the script gmap_build.

```
gmap_build -d dmelanogaster -D gmap-2016-09-23/gmapdb/
drosophila_melanogaster_genome_r6_P.fa
```

Then, GMAP was run with default parameters.

```
gmap –t 12 -A -f samse -d dmelanogaster -D gmap-2016-09-23/gmapdb/
dataset5.fastq > gmap_d5.sam 2> gmap_d5.log
```

On baseline Illumina dataset 0.1, GSNAP was run in addition to GMAP, also with default parameters.

```
gsnap -t 12 -A sam -d dmelanogaster -D gmap-2016-09-23/gmapdb/
dataset01.fastq > gsnap_d01.sam 2> gsnap_d01.log
```

Supplement note 3. Long read low error dataset

To better investigate the poor performance of some RNA mapping tools, we created an additional synthetic dataset containing long reads, but very few errors. It was used to determine which tools have trouble with longer reads and which with higher error rate.

Simulation parameters used with PBSIM are given below.

```
pbsim-1.0.3-Linux-amd64/Linux-amd64/bin/pbsim \
--data-type CLR --depth 2 \
--model_qc pbsim-1.0.3-Linux-amd64/data/model_qc_clr \
--length-mean 3080 \
--length-sd 2211 \
--length-min 50 \
--length-max 50000 \
--accuracy-mean 0.99 \
--accuracy-sd 0.10 \
--accuracy-min 0.95 \
--difference-ratio 47:38:15 \
../drosophila_melanogaster_transcriptome.fa
```

Supplement note 4. Additional simulated ONT MinION dataset using human chromosome 19

To further test the ability of aligners on simulated ONT data, we have created an additional synthetic dataset. It has been simulated using ONT MinION parameters and using human chromosome 19 as a reference. The results are show in the table below:

Table. Results on an additional ONT MinION dataset.

**Data set**	**Aligner**	**Aligned**	**Match rate**	**Correct**	**Hit all**	**Hit one**	**Split reads**	**Correct, split**	**Split hit all**	**Split hit one**
**ONT hChr19**	**STAR**	6.4%	**90.3%**	1.6%	5.58%	6.23%	4.42%	0.9%	3.64%	4.29%
	**BBMap**	63.3%	85.7%	7.8%	25.8%	61.7%	48.3%	3.7%	11.6%	47.5%
	**GMap**	**96.8%**	**90.3%**	**12.9%**	**77.1%**	**94.4%**	**82.2%**	**15.2%**	**63.8%**	**80.9%**

The results are similar to the ones on the simulated ONT MinION dataset in the main manuscript (dataset 8), with STAR having somewhat worse results. Since the results were as expected and since we didn't use a dedicated ONT MinION simulator, these results were not included in the main manuscript.

Supplement note 5. Description of metrics used for the evaluation

Due to the nature of the data different metrics were used to evaluate mapping tools on real and on synthetic datasets. Namely, for simulated data, the exact origin of each read is known and the corresponding alignment can be compared to it to examine how closely it matches. Read origins are determined from the simulation files generated by the simulation tool (PBSIM). For real data, the origins of reads are unknown and they are evaluated by comparing them to a set of known annotations to find out which annotations a read matches the most. The mapping quality is then calculated by comparing an alignment to that annotation.

Synthetic dataset evaluation.

The results for aligner evaluation on synthetic datasets is given in the Table 3 in the main manuscript. For each aligner and dataset, the following metrics are shown:

- Aligned – the percentage of reads for which the aligner produce an alignment, without examining whether the alignment is correct
- Match rate – the percentage of aligned bases that are equal to the corresponding bases on the reference. This is calculated across all alignments.
- Correct – the percentage of reads for which the beginning and the end match the beginning and the end of read origin, and for which the alignment also matches internal exon boundaries. To be considered correct, read beginning and end, as well as exon boundaries, must be within 5 bases from the origin. This metric enumerates reads that are considered correctly mapped.
- Hit all – the percentage of reads that overlap all exons from the read origin. Each overlap must be at least 5 bases to be considered valid.
- Hit one – the percentage of reads that overlap at least one exon from the read origin. Each overlap must be at least 5 bases to be considered valid.
- Split reads – the number of reads whose origin contains more than one exon.
- “Correct, split”, “Split hit all” and “Split hit one” – same as the metrics defined above, but calculated only for split reads.

Real dataset evaluation.

The results for aligner evaluation on real datasets is given in the Table 4 in the main manuscript. For each aligner and dataset, the following metrics are shown:

- Aligned – the percentage of reads for which the aligner produce an alignment, without examining whether the alignment is correct
- Match rate – the percentage of aligned bases that are equal to the corresponding bases on the reference. This is calculated across all alignments.
- No. expressed genes – number of genes overlapped by at least one read.
- Exon hit – the percentage of reads that overlap at least one exon.
- Contiguous alignment - the percentage of reads that correctly overlap a contiguous subset of exons belonging to a single annotation. The alignment must overlap a series of exons without skipping any inner exons and must align correctly to all inner exon boundaries within 5 bases. The alignment can skip exons from the start of annotation and from the end of annotation, as

long as the overlapped exons are contiguous.

A detailed description of the used metrics can be found at the RNAseqEval GtHub repository documentation (https://github.com/kkrizanovic/RNAseqEval).
